# Non-linear transformations of age at diagnosis, tumor size, and number of positive lymph nodes in prediction of clinical outcome in breast cancer

**DOI:** 10.1186/s12885-018-5123-x

**Published:** 2018-12-07

**Authors:** Carina Forsare, Martin Bak, Anna-Karin Falck, Dorthe Grabau, Fredrika Killander, Per Malmström, Lisa Rydén, Olle Stål, Marie Sundqvist, Pär-Ola Bendahl, Mårten Fernö

**Affiliations:** 1 Faculty of Medicine, Department of Clinical Sciences Lund, Oncology and Pathology, Lund University, Medicon Village Building 404, Scheelevägen 2, SE-223 81 Lund, Sweden; 2 Department of Pathology, Odense University Hospital, DK-5000 Odense, Denmark; 3 Department of Surgery, Helsingborg Hospital, SE-281 85 Helsingborg, Sweden; 4 Department of Pathology, Lund University, Skåne University Hospital, SE-221 85 Lund, Sweden; 5 Department of Haematology, Oncology and Radiation physics, Skane University Hospital, SE-221 85 Lund, Sweden; 6 Faculty of Medicine, Department of Clinical Sciences Lund, Division of Surgery, Skåne University Hospital, Lund University, SE-221 85 Lund, Sweden; 7 Department of Surgery and Gastroenterology, Skåne University Hospital, SE-205 02 Malmö, Sweden; 8 Department of Clinical and Experimental Medicine and Department of Oncology, Linköping University, SE-581 85 Linköping, Sweden; 9 Department of Surgery, County Hospital, SE-391 85 Kalmar, Sweden

**Keywords:** Breast cancer, Prognostic, Categorical, Continuous, Fractional polynomials, Splines

## Abstract

**Background:**

Prognostic factors in breast cancer are often measured on a continuous scale, but categorized for clinical decision-making. The primary aim of this study was to evaluate if accounting for continuous non-linear effects of the three factors age at diagnosis, tumor size, and number of positive lymph nodes improves prognostication. These factors will most likely be included in the management of breast cancer patients also in the future, after an expected implementation of gene expression profiling for adjuvant treatment decision-making.

**Methods:**

Four thousand four hundred forty seven and 1132 women with primary breast cancer constituted the derivation and validation set, respectively. Potential non-linear effects on the log hazard of distant recurrences of the three factors were evaluated during 10 years of follow-up. Cox-models of successively increasing complexity: dichotomized predictors, predictors categorized into three or four groups, and predictors transformed using fractional polynomials (FPs) or restricted cubic splines (RCS), were used. Predictive performance was evaluated by Harrell’s C-index.

**Results:**

Using FP-transformations, non-linear effects were detected for tumor size and number of positive lymph nodes in univariable analyses. For age, non-linear transformations did, however, not improve the model fit significantly compared to the linear identity transformation. As expected, the C-index increased with increasing model complexity for multivariable models including the three factors. By allowing more than one cut-point per factor, the C-index increased from 0.628 to 0.674. The additional gain, as measured by the C-index, when using FP- or RCS-transformations was modest (0.695 and 0.696, respectively). The corresponding C-indices for these four models in the validation set, based on the same transformations and parameter estimates from the derivation set, were 0.675, 0.700, 0.706, and 0.701.

**Conclusions:**

Categorization of each factor into three to four groups was found to improve prognostication compared to dichotomization. The additional gain by allowing continuous non-linear effects modeled by FPs or RCS was modest. However, the continuous nature of these transformations has the advantage of making it possible to form risk groups of any size.

**Electronic supplementary material:**

The online version of this article (10.1186/s12885-018-5123-x) contains supplementary material, which is available to authorized users.

## Background

Prognostic and treatment predictive factors in breast cancer (e.g. number of positive lymph nodes, age at diagnosis, tumor size, estrogen receptor (ER) and progesterone receptor (PgR), histological grade, and human epidermal growth factor receptor type 2 (HER2)) can predict clinical outcome and hence facilitate treatment choice [1, 2]. These factors can either be used individually or combined in indices such as e.g. the Nottingham Prognostic Index [3], CancerMath.net, Adjuvant! Online (http://cancer.lifemath.net) [4] or the St Gallen subtypes [5]. Prognostic factors are often continuous or measured on an integer-valued scale, but categorized for clinical decision-making. This application of prognostic factors in breast cancer has a long history dating back to the invention of the TNM classification system. Categorization of prognostic factors is intuitively appealing, since the clinically relevant question is often to select between a limited number of treatment modalities, but categorization of individual factors is not necessary for construction of useful prediction models [6]. On the contrary, numerous authors have discussed its negative consequences [7–9]. Categorization will in general lead to loss of information and hence lower power to detect true associations to prognosis and/or treatment response. To use dichotomized factors in prognostic models corresponds to assuming threshold effects and such effects are often biologically implausible. The use of multiple cut-points per factor, like e.g. T0 to T3 for tumor size in the TNM system is a step in the right direction, but how should cut-points be chosen for new prognostic factors? Optimal cut-offs, maximizing the prognostic value of a new factor in a specific dataset, will in general lead to biased effect estimates, even though methods have been designed to deal with this problem [10]. To avoid bias, pre-defined percentile-based cut-offs can be used, but different percentiles might be prognostically useful for different factors.

In survival analysis, the most commonly used model for analysis of multiple prognostic markers is the Cox proportional hazards regression model. In its simplest form, this model assumes constant, i.e. time independent, linear covariate effects on the log hazard scale or equivalently multiplicative effects on the hazard scale (proportional hazards). The log hazard is hence assumed to increase or decrease with the same constant additive factor for each step on the scale of the covariate, e.g. for each year of age at diagnosis of breast cancer. One way of relaxing this strong and often biologically unrealistic assumption of linear covariate effects is to use fractional polynomial (FP) transformations [11–14]. Transformations of this kind are useful when one wishes to preserve the continuous nature of the covariates in a regression model, but suspects that some of the effects may be non-linear. By taking non-linearity into account, more prognostic information will be extracted, which might have important clinical implications. A limited number of studies have addressed this question. Sauerbrei et al. have evaluated the use of FP transformations in Cox modelling of recurrence-free survival in a lymph node-positive breast cancer data set from the German Breast Cancer Study Group [15]. They conclude that analysis using FP transformations can extract important prognostic information which the traditional approaches may miss. More recently, Ejlertsen and co-workers have used FP transformations of age, tumor size, number of positive lymph nodes, and percentage of ER-positive nuclei, when developing a model for prediction of excess mortality after adjuvant endocrine therapy [16]. Compared to models with categorized predictors, models with FP transformations could better identify patients without excess mortality compared to the general population [16]. Another frequently used option is to model potential non-linear covariate effects on outcome using restricted cubic splines (RCS) [17, 18].

The primary aim of this study was proof of principle, i.e. to evaluate if accounting for non-linear effects of the three factors age at diagnosis, tumor size, and number of positive lymph nodes improves prognostication; factors which will be utilized also in the future after an expected implementation of gene expression profiling in clinical routine. Our hypothesis was that by keeping the predictors continuous as long as possible during the modeling process, prognostication would be improved.

## Materials and methods

### Derivation set

**Fig. 1:**
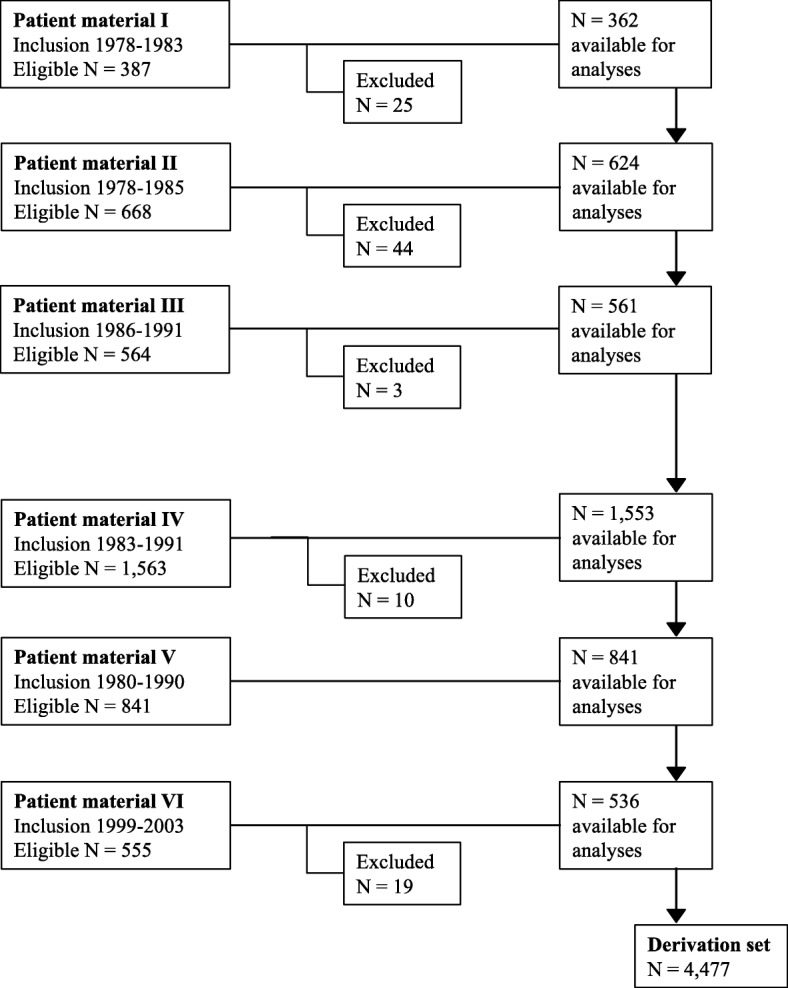
Flow diagram of the 4568 eligible patients, inclusion periods, patients excluded, and number of patients included for the different patient materials that constituted the derivation set. Patients were excluded due to missing information on follow-up, number of positive lymph nodes, and/or tumor size. One hundred and one patients were excluded and the final number of patients included was 4477

Included were 4568 women with primary breast cancer originating from four multicenter randomized controlled trials in stage II breast cancer (Patient materials I–IV; see below) and two prospectively followed cohorts (Patient materials V–VI; see below), more information in Additional file 1: Table S1. Patients were excluded due to missing information on follow-up, number of positive lymph nodes, and/or tumor size (Fig. 1), rendering 4477 patients included in the present paper. The endpoint was defined as distant recurrence-free interval (D-RFi) according to the DATECAN initiative (Definition for the Assessment of Time-to-event Endpoints in CANcer trials) [19] and the follow-up was restricted to ten years after diagnosis.

#### Patient material I

Patients were enrolled between 1978 and 1983 in a randomized controlled trial from the South Swedish Breast Cancer Group. The purpose of the trial was to evaluate the effect of chemotherapy (cyclophosphamide) and radiotherapy alone and in combination, in breast cancer women treated with modified radical mastectomy and axillary clearance. The original trial included 387 patients [20].

#### Patient material II

Patients were enrolled between 1978 and 1985 in a clinical study in the South Swedish Health Care Region, where postmenopausal patients were randomized to evaluate the effect of endocrine therapy (tamoxifen (TAM), one year) and radiotherapy alone and in combination. The original trial included 668 patients [21, 22].

#### Patient material III

Premenopausal patients were enrolled, between 1986 and 1991, in a randomized controlled trial with the aim to compare the effect of two years of TAM treatment versus no adjuvant systemic treatment (only eight patients received chemotherapy). The original trial included 564 patients enrolled in the South and South-East Swedish Health Care Regions [23].

#### Patient material IV

Postmenopausal patients were enrolled, between 1983 and 1991, in a randomized controlled trial launched by the Swedish Breast Cancer group of two versus five years of adjuvant TAM. The original trial included 1107 patients from the South Swedish Health Care Region [24]. The inclusion continued after the original paper was published, hence the greater number of 1553 patients included in the present paper.

#### Patient material V

The original study enrolled a consecutive series of 841 patients with primary breast cancer referred to Odense University Hospital, Denmark. Patients were enrolled between 1980 and 1990. The purpose was to investigate the prognostic value of estimating angiogenesis by Chalkley counting using a large population-based confirmatory study design [25].

#### Patient material VI

The original prospective observational study included 555 patients diagnosed with primary breast cancer from three hospitals in the South Swedish Health Care Region between 1999 and 2003. The purpose was to study the prognostic value of the presence of cytokeratin positive cells in bone marrow aspirates from the sternum [26].

The above patient materials (I–VI) constitute our derivation set, which has a median follow-up for D-RFi of 9.1 years.

### Validation set

#### Patient material VII

Between 1983 and 1999, a consecutive series of patients diagnosed with primary breast cancer in the Kalmar County, Sweden, were enrolled. The median follow-up time for distant recurrence-free survivors was 8.4 years.

### Statistical methods

The Kaplan-Meier method was used to estimate the primary endpoint, D-RFi [19], and the Cox proportional hazards model, stratified for patient material, for estimation of hazard ratios (HR) for groups formed by applying well-accepted pre-defined as well as percentile-based cut-offs. The relative effects of the factors in the Cox regression model are assumed to be constant, i.e. independent of time, assumptions which must be tested. Proportional hazards assumptions were checked graphically and by Schoenfeld’s test [27]. To avoid problems with non-proportional hazards, especially for tumor size, the follow-up was restricted to the first ten years of follow-up after diagnosis. This restriction has the additional advantage that the median follow-up in the included patient materials will be about the same. The statistical analysis software Stata 15.0, 2017 (StataCorp, College Station, TX, USA) was used for statistical calculations. Whenever applicable, the REMARK recommendations for reporting of tumor marker studies were followed [28, 29].

Continuously varying non-linear effects on the log hazard were modeled using FP transformations [12, 13] in Cox regression models. To avoid over-fitting, a function selection procedure based on a closed test procedure was used which adds flexibility, i.e. extra polynomial terms, only if the model fit improves significantly at the chosen overall significance level after adjustment for multiple testing [13]. The multivariable FP procedure (MFP; default settings in Stata), which is an extension of the function selection procedure based on FPs, was used to derive an FP-based prognostic index based on the three originally continuous or integer valued predictors [13]. For simplicity, we will henceforth use the term continuous for both types of scales – the truly continuous used for age and tumor size and the integer valued used for number of positive nodes. In this paper significance testing was applied only during model selection within the FP procedure and then the alpha level was set at the default value 0.05.

Separate Cox-models were fitted for each of the three covariates in the derivation set. For each model, the MFP procedure was used to automatically select the best fitting transformation(s). Thereafter, predicted relative hazards were calculated for each factor and patient. These predictions were plotted versus each factor to graphically describe the functional form of the relationships. The following reference values were chosen for calculation of relative hazards: age 35 years, tumor size 20 mm, and 0 positive lymph nodes. 95% confidence intervals (CI) for the relative hazards were calculated using bootstrap resampling. Briefly, the model selection procedure and the corresponding calculation of relative hazards was replicated for 1000 bootstrap samples and the lower and upper limits were chosen as the 2.5 and 97.5% percentiles, respectively, for each covariate and observed value. The distribution of each factor is also shown in these graphs, both as dots along a line and as a kernel density estimate. The default options in Stata were used for kernel density estimation.

Non-linear covariate effects were also modeled using RCS. In brief, for each covariate, *k* so-called knots, was chosen which uniquely define *k-1* polynomial transformations of the covariate. The definition of these transformations guarantees that any linear combination of the *k-1* spline variables will be linear before the first knot, a piecewise cubic polynomial between adjacent knots, and linear again after the last knot. To avoid over-fitting, we decided to use five knots located at the 5th, 27.5th, 50th, 72.5th and 95th percentiles as recommended by Harrell [17]. This definition was found to work for age and tumor size. For number of positive lymph nodes, a variable with almost 40% zeros, we chose to place the five knots at 1, 2, 3, 4 and 10 positive nodes.

The statistical models developed in the derivation set were tested in the validation set. Briefly, the transformations of the covariates, and the corresponding weights from estimation in the derivation set, were applied to calculate the value of a prognostic index for each patient in the validation set. Patients were then divided into risk groups based on this index to assess the discrimination in the validation set. A proper validation should assess both discrimination and calibration [30], but calibration could not be reliably assessed in the present study due to differences in the distribution of prognostic factors, treatments, calendar periods and length of follow-up.

Different measures of predictive performance and model fit for Cox proportional hazards model have been suggested in the literature [30]. In the present paper, we have chosen Harrell’s Concordance index (C), which is a generalization of the area under the receiver operating characteristic curve (AUC) for survival data [31]. It is defined as the fraction of all evaluable pairs of patients for which the patient with the best observed survival also has the lowest predicted hazard [32]. Hence, the C-index will be 0.500 for a useless model and 1.000 for a model with optimal fit to the data.

## Results

### Patient and tumor characteristics

During 10 years of follow-up, distant recurrences were recorded for 1315 of the 4477 patients (29%) in the derivation set. Median age was 60 years (range: 25–93), median tumor size 22 mm (range: 1–120 mm), and 40% were lymph node-negative. Endocrine treatment alone was given to 59%, chemotherapy alone to 10%, and chemo-endocrine therapy to 2% of the patients. Only five patients were given anti-HER2 treatment. Clinical and histopathological characteristics for the patients in the derivation set and in the separate patient materials are shown in Table 1 and Additional file 1: Table S1, respectively.

**Table 1:** Patient and tumor characteristics for the derivation and validation sets

Factor	Derivation set	Validation set
*No of patients*	4477	1132
*Distant recurrences* ^*a*^	1315 (29)^b^	289 (26)
*Age* median, years	60	64
*Age* range, years	25–93	28–99
Age < 35	69 (2)	11 (1)
Age 35–50	1023 (23)	202 (18)
Age > 50	3385 (76)	919 (81)
*Tumor size* median, mm	22	20
*Tumor size* range, mm	1–120	1–160
T1 (≤20 mm)	1942 (43)	590 (52)
T2 (21–50 mm)	2460 (55)	506 (45)
T3 (> 50 mm)	75 (2)	36 (3)
*Lymph nodes* median	1	0
*Lymph nodes* range	0–47	0–23
Negative	1783 (40)	659 (58)
1–3 positive	1781 (40)	305 (27)
4–9 positive	649 (14)	127 (11)
≥ 10 positive	264 (6)	41 (4)
*Adjuvant medical treatment*		
Endocrine therapy	2662 (59)	673 (59)
Chemotherapy	460 (10)	52 (5)
Chemo-endocrine	74 (2)	38 (3)
None	1279 (29)	369 (33)
Missing	2	0

^a^Follow-up truncated at 10 years

^b^Numbers in parentheses are percentages

### Univariable analyses

#### Dichotomized predictors

**Fig. 2:**
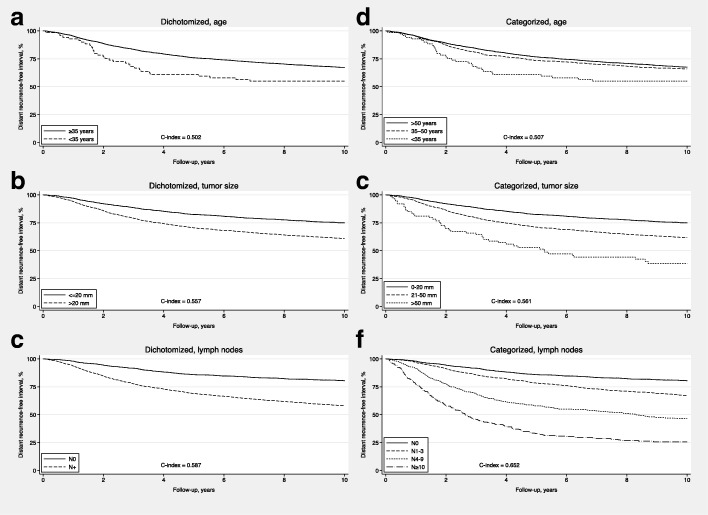
Univariable analyses with the predictors dichotomized (< 35 vs. ≥35 years, > 20 vs. ≤20 mm, and positive vs. negative lymph nodes; 2**a**–**c**) and categorized accordingly: age at diagnosis in three categories (> 50, 35–50, and < 35 years at diagnosis), tumor size in three (T1, T2, and T3), and lymph nodes in four categories (N0, N1–3, N4–9, and *N* ≥ 10; 2**d**–**f**)

Age at diagnosis, when categorized into two groups (< 35 vs. ≥35 years), was associated to D-RFi, HR = 1.49 (95% CI: 1.04–2.13, C-index: 0.502). The corresponding HR for tumor size (> 20 vs. ≤20 mm) was 1.65 (95% CI: 1.47–1.86, C-index: 0.557), and for positive vs. negative lymph nodes 2.40 (95% CI: 2.11–2.73, C-index: 0.587). The respective Kaplan-Meier estimates of D-RFi are shown in Fig. 2 a-c.

#### Categorized predictors in three or four groups

Age was categorized in three groups (> 50, 35–50, and < 35 years at diagnosis), tumor size also in three groups (≤20 mm (T1), 21–50 mm (T2), and > 50 mm (T3)), and number of positive lymph nodes in four groups (no positive nodes (N0), 1–3 positive (N1–3), 4–9 positive (N4–9), and ≥ 10 positive lymph nodes (*N* ≥ 10)). The addition of an extra cut-off at 50 years and 50 mm for age and tumor size, respectively, lead to only minor increases in C-indices, from 0.502 to 0.507 for age and from 0.557 to 0.561 for tumor size. For number of positive lymph nodes, adding two additional cut-offs at 4 and 10 positive nodes lead to a more pronounced increase, from 0.587 to 0.652. The associations between these categorized variables and D-RFi are further illustrated in Fig. 2 d, e and f and in Additional file 2: Table S2.

#### Continuous predictors

Initial FP-modeling revealed a non-monotonic relationship between relative hazard of distant recurrences and tumor size. However, a sensitivity analysis showed this unexpected pattern to be caused by a small fraction (12/4477; 0.3%) of very small tumors (≤2 mm) with more distant recurrences than expected. After reviewing the original pathology reports, the registered tumor size for four of these patients was found to be wrong and was therefore corrected to sizes ranging from 20 to 25 mm. All results presented in this paper correspond to the corrected database.

**Fig. 3:**
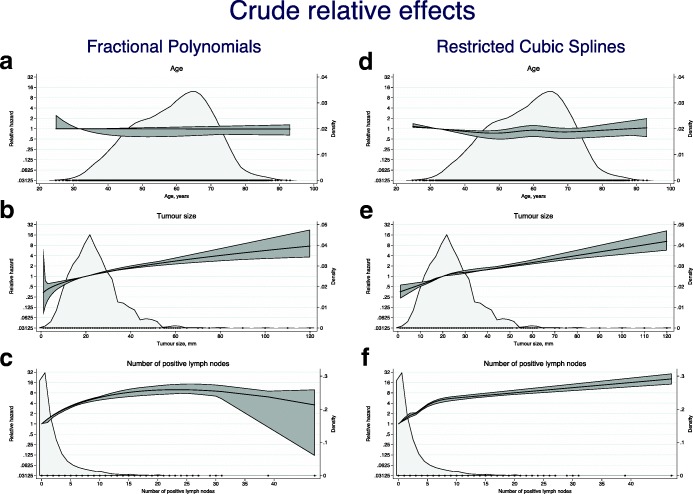
Univariable analysis of non-linear effects for age, tumor size, and number of positive lymph nodes using MFP (3**a**–**c**) and RCS (3**d**–**f**). For each factor X, an estimate of the relative hazard is shown as a function of X. The estimate is based on a Cox model with fractional polynomial transformation of X. A reference value was chosen for each factor (35 for age, 20 for tumor size, and zero for number of positive lymph nodes). The relative hazard for this value will be 1.00 per definition. For other values of each factor the relative hazard will be an estimate with a corresponding 95% CI shown as a band around the point estimate. The shaded area in the background is a kernel density estimate of the distribution of each factor and the dots represent the values observed

In the final FP-analyses, non-linear effects were detected for tumor size and number of positive lymph nodes, but not for age, see Fig. 3a-c. The C-index for age, for which the linear identity transformation was chosen by the MFP procedure, was 0.513. A square root transformation provided best fit for tumor size, C-index 0.594, whereas a linear combination of two polynomial terms provided the best fit, according to the MFP procedure, for number of positive lymph nodes leading to a C-index of 0.665. A sensitivity analysis excluding the patient with the highest number of positive lymph nodes (*N* = 47), did not lead to a final model with fewer degrees of freedom.

Similar results were found with restricted cubic splines (Fig. 3d-f), except that the sensitivity to outliers was better handled by the restriction to linearity outside the most extreme knots. The corresponding C-indices were 0.516, 0.594, and 0.665 for age, tumor size and number of positive lymph nodes, respectively.

### Multivariable analyses

#### Model based on dichotomized predictors

The C-index for this model was 0.628, which should be compared to the C-indices for the corresponding univariable models, range 0.502–0.587. The estimated adjusted effects for the three factors were HR = 1.54 for age at diagnosis (< 35 vs. 35 ≥ years), 95% CI: 1.07–2.21, HR = 1.85 for tumor size (> 20 vs. ≤20 mm), 95% CI: 1.65–2.08, and HR = 2.60 for positive vs. negative lymph nodes, 95% CI: 2.28–2.96.

#### Model based on categorized predictors in three or four groups

Cox regression with age at diagnosis and tumor size in three categories, and number of positive lymph nodes in four categories further improved risk stratification, with a corresponding C-index of 0.674. For comparison with models based on FPs and RCS, see below. The predicted relative hazards, with 31 distinct values corresponding to the 31 actually observed of the 36 possible combinations (3x3x4) of the three categorized factors, were divided into four groups aiming at the 16th, 50th, and 84th percentile, following the recommendation by Royston and Altman [30]. The closest possible fit to this recommendation for the present dataset resulted in the 15th, 44th, and 84th percentiles with 10-year D-RFi (95% CI): 88% (86–91), 75% (72–77), 65% (62–67), and 36% (33–40), respectively.

#### Models based on continuous predictors

Multivariable FP (MFP) and RCS transformations improved the C-index further, to 0.695 and 0.696, respectively. Second degree FPs, i.e. linear combinations of two polynomial transformations, were chosen by the MFP procedure for both tumor size and number of positive lymph nodes. However, a sensitivity analysis excluding the patient with the highest number of positive lymph nodes, (*N* = 47), revealed that the second polynomial term for number of positive nodes in the multivariable model was driven by this outlier. A single polynomial term, a log-transformation, for this variable would have been sufficient if this patient had been excluded. The C-index for this less complex MFP-model was 0.692.

Furthermore, the predictions from these two models, MFP and RCS, were divided into the four percentile-based subgroups mentioned above. For MFP, the 10-year D-RFi-Figures (95% CI) were 90% (87–92), 76% (74–78), 62% (59–65), and 36% (32–40), respectively, and for RCS: 89% (86–91), 76% (74–79), 62% (59–65), and 35% (32–39), respectively.

### External validation

During 10 years of follow-up, distant recurrences were recorded for 289 of the 1132 patients in the validation set (Material VII). Median age was 64 years (range: 28–99), median tumor size 20 mm (range: 1–160 mm), and 58% were lymph node-negative. Endocrine treatment only was given to the same fraction of patients as in the derivation set, 59%, whereas chemotherapy only was less frequently administered, just 5%. Chemo-endocrine treatment was given to 3% of the patients (Table 1).

Four multivariable prediction models fitted in the derivation set were evaluated in the validation set (*N* = 1132); the models with dichotomized and categorized predictors (≥2 cut-offs), the MFP model and the RCS model. For each model, the same predictor transformations were applied in the validation set as in the derivation set and the weights estimated in the derivation set, i.e. the estimated log relative hazards, were used to calculate the values for the prognostic indices (PIs) for the patients in the validation set. These PIs rank the patients in the validation set from lowest to highest risk based on external data. Hence, unbiased C-indices could be calculated. The validation C-index for the model with dichotomized predictors was 0.675. As expected, the discrimination in the validation set turned out to be better for the model with categorized predictors, C = 0.700, but the extra gain by allowing FP- or RCS-transformations was almost negligible, C-indices 0.705 and 0.701, respectively.

**Fig. 4:**
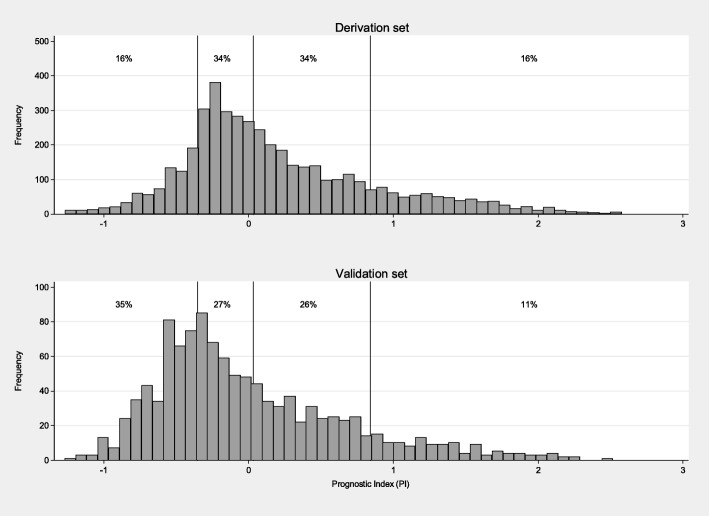
Histograms showing the distributions of the prognostic indices (PI) corresponding to the final MFP model in the derivation set and the validation set. The indices for both sets have been centered by subtracting the mean PI in the derivation set. The vertical lines represent the 16th, 50th, and 84th percentiles of the PI-distribution in the derivation set – cut-offs which identify groups of different relative sizes in the validation set

For all the four models, the distribution of the PI was found to be shifted to the left, i.e. towards lower risk, in the validation set compared to the derivation set. This is in agreement with the patient characteristics presented in Table 1. Most notably, the fraction of lymph node-negative patients is higher in the validation set (58% vs. 40%). As an example, histograms of the PI-distributions in the derivation and validation datasets for the MFP-model are shown in Fig. 4.

**Fig. 5:**
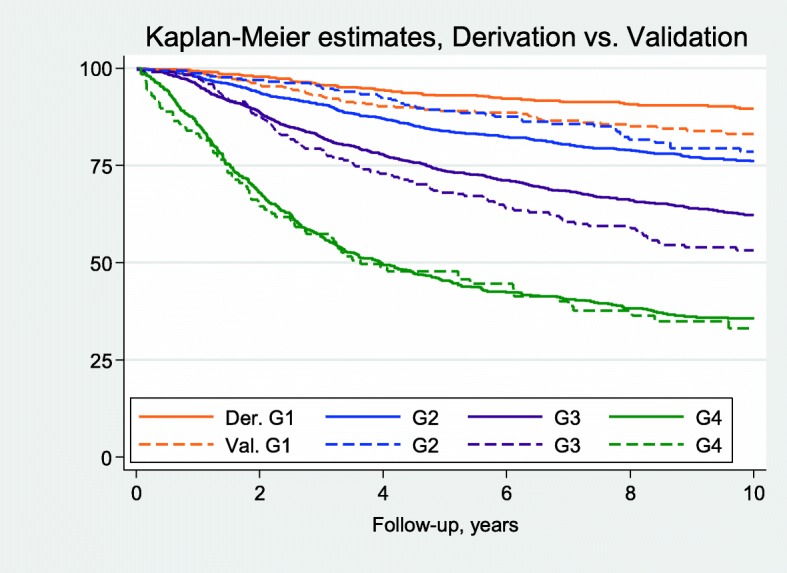
Kaplan-Meier estimates of distant recurrence-free interval (D-RFi) for risk groups G1 to G4 formed by categorization of the prognostic index (PI) for the MFP model in the derivation set at the 16th, 50th and 84th percentiles. Solid lines were used for estimates in the derivation set and dashed lines for the corresponding estimates in the validation set. Note that the actual values of the PI at the cutoffs in the derivation set were used as cutoffs for the PI in the validation set, leading to different relative sizes of the four groups in the two datasets. Abbreviations: Der. = Derivation set, Val. = Validation set

The prognostic discrimination in the derivation and the validation set, respectively, of the models based on FP- and RCS-transformed predictors was further analyzed by calculation of HR:s for the four risk groups; G1 (reference), G2, G3, and G4, defined by cut-offs at the 16th, 50th, and 84th percentiles of the PIs in the derivation set, see Table 2. The corresponding risk groups, formed by applying the actual values of the PIs in the derivation set as cut-offs for the PIs calculated in the validation set lead to risk groups of other relative sizes in the validation set. Instead of 16/34/34/16 for G1/G2/G3/G4, the percentages in the four risk groups turned out to be 35/27/26/11 and 34/29/25/12 for MFP and RCS, respectively, again reflecting a shift towards lower risk in the validation set. The HR:s in the column ‘Validation’ of Table 2 reflect the discrimination of the prognostic models in an independent patient material. Briefly, the results from the FP- and RCS-modelling were comparable and as expected, the relative effect estimates were smaller, i.e. closer to 1.00, when the models fitted in the derivation set were applied to the validation set. The corresponding Kaplan-Meier estimates for the MFP-based model, see Fig. 5, show that the discrimination between G1 and G2 in the validation set is poor. However, G3 and G4 are well separated in the validation set and these risk groups are also well separated from G1 and G2. Note also that the calibration of the high-risk group is good, reflected by the almost completely overlapping survival estimates for the two data sets.

**Table 2:** Hazard ratios from analysis of D-RFi for Cox-models with MFP- and RCStransformations of the predictors

Model	Derivation	Validation
*MFP*	*HR (95% CI)*	*HR (95% CI)*
G2 vs. G1	2.46 (1.89–3.20)	1.16 (0.78–1.72)
G3 vs. G1	4.28 (3.31–5.54)	3.36 (2.43–4.65)
G4 vs. G1	10.5 (8.13–13.7)	6.92 (4.85–9.86)
*RCS*		
G2 vs. G1	2.36 (1.81–3.06)	1.46 (1.00–2.13)
G3 vs. G1	4.15 (3.23–5.34)	3.10 (2.20–4.35)
G4 vs. G1	10.4 (8.06–13.4)	7.97 (5.57–11.4)

For each method of covariate transformation, the prognostic index (PI) in the derivation set was categorized at the 16th, the 50th, and the 84th percentiles forming four risk groups named G1 to G4. The parameter estimates from the derivation set for MFP and RCS were used to calculate the PIs for each patient in the validation set. Each of these two indices was thereafter categorized into four groups using the percentile based cut-off values from the derivation set

## Discussion

Using a large cohort comprising 5609 patients with D-RFi as primary endpoint, we detected non-linear relationships to the relative hazard of distant recurrences for tumor size and number of positive lymph nodes, but not for age at diagnosis. These findings were, however, found to be of minor importance for prognostication of 10-years D-RFi in the multivariable modeling with FP transformations, since, in contrast to what we expected, only a modest increase in C-index was obtained for the model based on continuous variables compared to the model with categorized predictors. In the derivation set, a model with age and tumor size in three categories and number of positive lymph nodes in four categories, was considerably better than the corresponding model applying dichotomized variables (C-index: 0.674 vs. 0.628). These findings support the way tumor size and number of positive lymph nodes are used in the clinical decision-making today. The putative non-linear effects of these variables seem to be sufficiently captured by increasing the number of cut-offs from one to two or three. The drawback is information loss and that categorization might lead to tied predictions for large groups of patients, prohibiting the possibility to create risk groups of any size desired. Similar results were obtained in the validation set. Furthermore, the HR:s comparing the prognosis in the four groups, based on the 16th, 50th, and 84th percentile of the prognostic index derived from the final MFP model were similar in the derivation and validation sets. The relative effect estimates were smaller when the models fitted in the derivation set were applied to the validation set. This could be explained by over-fitting to the derivation set.

In contrast to previous studies, [33, 34] we found no effect of age on D-RFi. This could be explained by that 33% (23/69) of the patients below the age of 35 have been treated with adjuvant chemotherapy compared to only 12% (511/4406) of the patients above 35 years. The importance of chemotherapy for the association between age and prognosis has been demonstrated by others [33, 34]. Another possible explanation for the non-existing age trend in the present study is that the fraction of patients below 35 years is lower in this study than previously reported for population based breast cancer series, diluting the power to detect a trend.

In contrast to our results, Ejlertsen and co-workers have shown that FP transformation outperformed the predictions based on categorized variables [16]. This may be explained by that they also included the percentage of ER-positive nuclei in their algorithm and furthermore used a population-based and more homogenous derivation cohort of 6529 postmenopausal high-risk patients, all receiving five years of adjuvant endocrine therapy. Also, the study by Sauerbrei and colleagues concluded that FP extracted more prognostic information in a study only including patients with lymph node-positive breast cancer (*N* = 686; [15]).

We have used both MFP- and RCS-transformations to model potentially non-linear relationships to prognosis for the factors age at diagnosis, tumor size and number of positive lymph nodes. The results, as measured by the C-indices and the functional form of the relationships, were strikingly similar. These transformation methods have advantages and disadvantages, as discussed by Royston and Sauerbrei [13]. FPs are more sensitive to outliers, but this can be handled for example by restricting the degrees of freedom for each factor. A single patient with 47 positive lymph nodes, which was the most extreme value observed in the derivation set, altered the shape of the estimated relationship. A sensitivity analysis revealed that the final prognostic model suggested by the MFP procedure had fewer degrees of freedom when this patient was excluded. RCS, on the other hand can lead to over-fitting [13] especially if many knots are used. The integrated automatic selection of variables and functional forms of these, implemented in the MFP procedure, gives some protection against over-fit, but to avoid capturing too much of nuances in the data set used for estimation, incorporation of prior knowledge should also be considered during the statistical modeling. Another, alternative modeling strategy is artificial neural networks (ANN), which was recently applied to a dataset, which largely overlaps with the derivation set in the present paper [35]. The performance of ANN and Cox models were almost identical.

In an initial FP-modeling step, we revealed a non-monotonic relationship between the relative hazard of distant recurrences and tumor size. This was caused by incorrect values of tumor size for four patients in the very small subset of patients with tumors less or equal to 2 mm. This finding highlights the importance of the quality of the data. We have not been able to perform a complete examination of all figures in the database, but the study of Rydén et al., demonstrate good agreement for parts of the patient material included in the present study [36].

One limitation with the present study is that only the three factors age at diagnosis, tumor size, and number of positive lymph nodes are included. A clinically applicable model should include all prognostic factors in use, i.e. according to current guidelines also ER, PgR, HER2, and histological grade [2, 37]. Unfortunately, we did not have complete and standardized information for these additional factors. However, in an expected future situation, when different gene profiles have replaced single biomarker analyses, age, tumor size and number of positive lymph nodes will most likely still be included in clinical routine management of breast cancer patients, and therefore the results obtained with these three factors in the present work, should retain their value. Another limitation is that the derivation dataset is not population based, but rather consists of patients included in randomized controlled trials and well-defined cohorts from different geographical areas and time periods. A strength, on the other hand, is that the models fitted in the derivation set were successfully validated in an independent dataset, even though the validation set had a higher proportion of N0 compared to the derivation set. This suggests that the results are generalizable and robust. The discrimination was found to be better for high-risk patients than for patients whose prognostic factors indicated lower risk. Differences between the derivation set and the validation set in this study can explain the sub-optimal performance of the prediction models, but perfectly matching dataset are hard to find and it is desirable that the performance of prognostic models is good also in datasets with slightly different characteristics. Future studies aiming at clinically useful models, should be thoroughly assessed for both discrimination and calibration in external datasets, see [30] for details.

In conclusion, categorization of age at diagnosis, tumor size, and number of positive lymph nodes into three to four groups was found to improve prognostication compared to dichotomization. The additional gain by allowing continuous non-linear effects modeled by FPs or RCS was modest – a finding in line with the famous statistician John Tukey’s advice of parsimony [38].

## Additional files

Additional file 1: **Table S1.** Patient and tumor characteristics for the different patient materials in the derivation set. (PDF 78 kb)

Additional file 2: **Table S2.** Categorized predictors in three or four groups. (PDF 66 kb)

## Abbreviations

ANN: Artificial neural network
AUC: Area under the receiver operating characteristic curve
C: Concordance index
CI: Confidence interval
D-RFi: Distant recurrence-free interval
ER: Estrogen receptor
FP: Fractional polynomial
HER2: Human epidermal growth factor receptor 2
HR: Hazard ratio
MFP: Multivariable fractional polynomial
N0: No positive lymph nodes
N1–3: 1–3 positive lymph nodes
N4–9: 4–9 positive lymph nodes
N ≥ 10: ≥ 10 positive lymph nodes
PgR: Progesterone receptor
PI: Prognostic index
RCS: Restricted cubic spline
T1: Tumor size ≤20 mm
T2: Tumor size 21–50 mm
T3: Tumor size > 50 mm
TAM: Tamoxifen
TNM: Tumor node metastasis
