# Supplementary material for: Non-linear transformations of age at diagnosis, tumor size, and number of positive lymph nodes in prediction of clinical outcome in breast cancer
Source: BMC Cancer. 2018 Dec 7;18:1226. doi: 10.1186/s12885-018-5123-x (PMC6286551; doi:10.1186/s12885-018-5123-x)
Supplement: Supplementary file 1 — Table S1. Patient and tumor characteristics for the different patient materials in the derivation set. (PDF 78 kb) [file 12885_2018_5123_MOESM1_ESM.pdf]

Supplement Table 1. Patient and tumor characteristics for the different patient materials in the derivation set.

Factor	I[a]	II[a]	III[a]	IV[a]	V[a]	VI[a]
No of patients	362	624	561	1,553	841	536
Distant recurrences [b]	125	237	233	382	251	87
Age **median, years**	47	63	45	65	59	58
Age **range, years**	27–63	34–71	25–58	43–86	24–93	25-88
Age <35	12	1	33	0	16	7
Age 35–50	233	4	438	26	220	102
Age >50	117	619	90	1,527	605	427
Tumor size						
T1 (≤20 mm)	148	257	207	548	425	357
T2 (21-50 mm)	212	367	351	998	361	171
T3 (>50 mm)	2	0	3	7	55	8
Lymph nodes						
Negative	125	258	160	518	403	319
1-3 positive	165	251	274	671	279	141
4-9 positive	50	82	97	277	102	41
≥10 positive	22	33	30	87	57	35
Adjuvant medical treatment						
Endocrine therapy	0	419	270	1,553	139	281
Chemotherapy	236	0	4	0	163	57
Chemo-endocrine	0	0	4	0	32	38
None	126	205	283	0	507	158
Missing	0	0	0	0	0	2

[a]Patient material I [20], II [21, 22], III [23], IV [24], V [25], and VI [26].

[b]Number of distant recurrences is for 10 years follow-up time.
