# Supplementary material for: Non-linear transformations of age at diagnosis, tumor size, and number of positive lymph nodes in prediction of clinical outcome in breast cancer
Source: BMC Cancer. 2018 Dec 7;18:1226. doi: 10.1186/s12885-018-5123-x (PMC6286551; doi:10.1186/s12885-018-5123-x)
Supplement: Supplementary file 2 — Table S2. Categorized predictors in three or four groups. (PDF 66 kb) [file 12885_2018_5123_MOESM2_ESM.pdf]

Supplement Table 2. Categorized predictors in three or four groups.

Factor	HR (95% CI)
Age at diagnosis	
<35 years vs. >50 years	1.39 (0.95–2.02)
35–50 years vs. >50 years	0.89 (0.75–1.06)
Tumor size	
T2 vs. T1	1.60 (1.42–1.80)
T3 vs. T1	3.53 (2.56–4.88)
Lymph nodes	
N1–3 vs. N0	1.66 (1.44–1.91)
N4–9 vs. N0	3.50 (2.98–4.10)
N≥10 vs. N0	7.23 (6.02–8.68)
